# Contrast-induced nephropathy prediction following ST-elevation myocardial infarction: Missing links

**DOI:** 10.34172/jcvtr.2023.31706

**Published:** 2023-03-16

**Authors:** Rohan Magoon, Brajesh Kaushal

**Affiliations:** ^1^Department of Anaesthesi, Atal Bihari Vajpayee Institute of Medical Sciences (ABVIMS) and Dr. Ram Manohar Lohia Hospital, Baba Kharak Singh Marg, New Delhi-110001, India; ^2^Department of Anaesthesia, Gandhi Medical College and Hamidia Hospital, Bhopal 462001, Madhya Pradesh, India

## Dear Editor,

 The study by Ceylan and Yildirim outlining H_2_FPEF score as independent predictor of contrast-induced nephropathy (CIN) in ST-elevation myocardial infarction (STEMI) patients undergoing percutaneous coronary intervention (PCI), emerges as an exemplary research work.^1^ Nonetheless, considering the relevance of the research subject, additional insights into the topic would certainly interest the Journal readership.

 The authors retrospectively analyze the data of 355 patients, wherein one misses a comparative account of the serum albumin (SA) levels in the cohort developing CIN (n = 63) as opposed to those without CIN (n = 292).^1^ The importance of the former is heralded by the Wiedermann et al meta-analysis suggesting a causal association between hypoalbuminemia and acute kidney injury (AKI), emanating from a total of 43 studies and 68,262 subjects.^2^ Notably so, the meta-analysis included 8 studies with 4,344 patients combined from either cardiac surgical or PCI settings.^2^

 More importantly, Murat et al retrospectively delineate significantly lower SA in their acute coronary syndrome (ACS) patients with contrast-induced AKI, or CI-AKI (n = 107) compared to those with normal renal function following PCI (n = 783) (3.52 ± 0.40 v/s 3.94 ± 0.39 mg/dL, *P* < 0.001).^3^ Subsequent to a multivariate analysis, the group highlights SA as an independent CI-AKI predictor (OR; 95% CI: 0.177; 0.080-0.392, *P* < 0.001) in a research scenario demonstrating peculiar similarities to the Ceylan and Yildirim study.^1,3^

 Ahead of the well-known pro-inflammatory links of both hypoalbuminemia and AKI, Wei et al elucidate a pivotal role of nutritional status in determining the eventual risk to contrast-induced renal injury in an elderly subset undergoing PCI.^2-5^ Indeed, the dual relationship of SA with malnutrition and inflammation cannot be overemphasized, particularly in advanced age.^3,5^ Moreover, whilst the inclusion of SA could have augmented the contextual research lucidity, the optimal CIN-predictive cut-off of the H_2_FPEF score might also require further consolidation given the recent literature in the subject (H_2_FPEF cut-off of 2.5 predicting CIN in ACS patients with 79.8% and 64.1% sensitivity-specificity in Ozbeyaz et al study vis-à-vis a CIN-predictive cut-off of 1.5 emanating from the Ceylan and Yildirim study and demonstrating a sensitivity-specificity of 64.0% and 72.1% in an exclusive STEMI cohort).^1,6^

## Competing Interests

 Nothing to declare.
